# Diffuse Large B‐Cell Lymphoma With Membranous Proliferative Glomerulonephritis as the First Manifestation: A Case Report

**DOI:** 10.1002/ccr3.70353

**Published:** 2025-04-03

**Authors:** Hongrui Xiao, Lei Ye, Fenjuan Zhang

**Affiliations:** ^1^ Department of Hematology Lishui Municipal Central Hospital Lishui Zhejiang China

**Keywords:** case report, diffuse large B‐cell lymphoma, membranous proliferative glomerulonephritis, nephrotic syndrome

## Abstract

Clinicians should investigate the causes of kidney disease in patients with poor therapeutic effects. Lymphoma should be considered in patients with renal damage combined with anemia, elevated globulin, elevated immunoglobulin, and lymphadenopathy. Promptly perform renal biopsy, lymph node biopsy, and blood and bone marrow specimen examination to avoid misdiagnosis and delayed treatment.

## 1 Introduction

Diffuse large b‐cell lymphoma (DLBCL) is a highly invasive lymphoma, which is often associated with extranodal organ involvement. However, its combined renal involvement is rare [1, 2]. There is a link between lymphoma and kidney disease. Lymphoma can directly infiltrate the patient's kidneys or indirectly damage the kidneys in some way. To date, a variety of mechanisms have been proposed to explain the link between lymphoma and kidney disease [3]. Lymphoma can directly affect the kidney, such as lymphoma occupying compression of urinary tract, lymphoma compression of renal artery and tumor cells can also directly infiltrate the kidney. Some types of lymphoma can cause cellular immune dysfunction, produce abnormal cytokines and autoantibodies, leading to increased glomerular permeability and kidney disease. Some types of lymphoma can also secrete monoclonal IgM, which leads to glomerular hair cell vascular damage and kidney disease. A retrospective study of 20 cases of non‐Hodgkin's lymphoma‐associated kidney damage in China found that chronic lymphocytic leukemia/B‐cell lymphoma accounted for a higher proportion [4]. The most common histological changes of renal biopsy were glomerular membrane hyperplasia, followed by crescent formation.

The patient was initially diagnosed with nephrotic syndrome (NS) as the first symptom. Renal biopsy showed membranous proliferative glomerulonephritis without common clinical manifestations of lymphoma. This article suggests that clinicians should pay attention to the etiological diagnosis of NS. For NS with repeated symptoms and poor treatment effect, the etiology should be found in time. Lymphoma should be considered in patients with renal damage with abnormal blood routine (lymphocyte count) and lymph node enlargement. Lymph node biopsy, renal biopsy, blood, and bone marrow specimens should be completed in time for detailed examination. We report the clinical data of a case of DLBCL with membranous proliferative glomerulonephritis as the first manifestation, in order to attract clinical attention and avoid missed diagnosis and misdiagnosis.

## 2 Case History

A 65‐year‐old female patient was admitted to our hospital on April 5, 2022, for eyelid and leg edema of 10 months in duration. The patient was previously healthy and had no significant past medical history, family history, or NSAID or other noteworthy drug use. She was initially examined, and her urine test results were as follows: urine occult blood, protein 3+, 24‐h urine protein 8320.45 mg. The biochemical values were as follows: albumin, 18.6 g/L; and creatinine, 260 μmol/L. Consequently, the patient was admitted to the ward for further examination and treatment.

## 3 Methods

**FIGURE 1:**
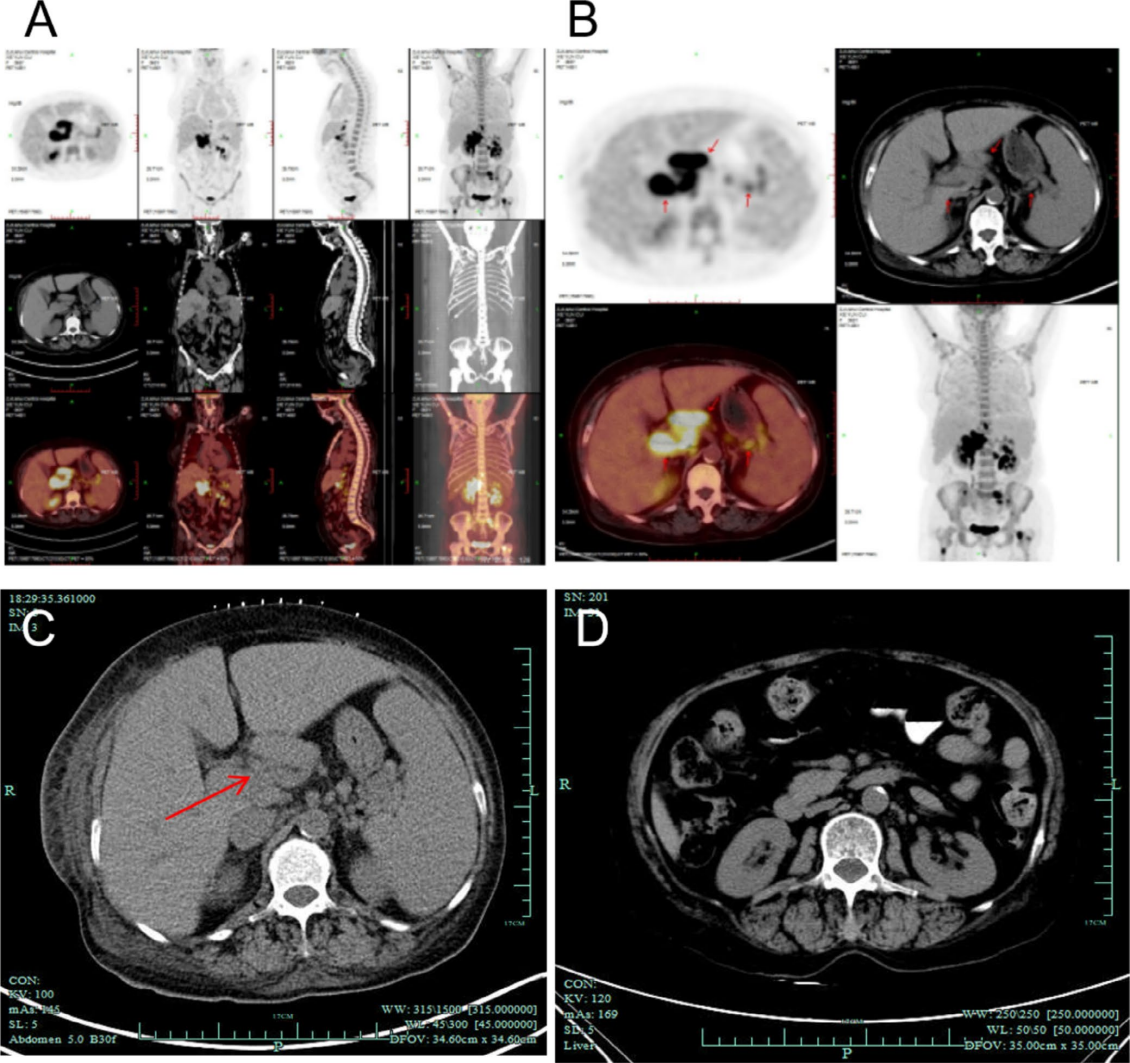
(A, B) PET–CT examination revealed multiple enlarged lymph nodes around the inferior vena cava, hepatic hilum, retroperitoneum and splenic hilum, as well as increased FDG metabolism. (C, D) The size of lymph nodes in abdominal CT was changed before and after R‐CHOP treatment.

**FIGURE 2:**
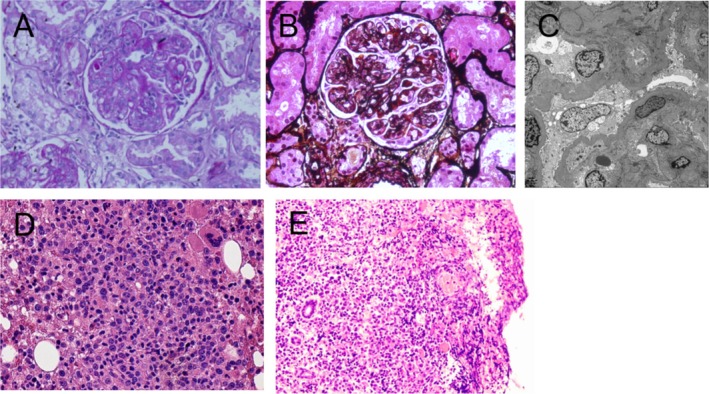
Biopsy pathology: (A) Periodic Acid‐Schiff stain of left renal biopsy (magnified ×40). (B) Periodic acid‐silver methenamine stain of left renal biopsy (magnified ×40). (C) The electron microscopic images of left renal biopsy. (D) HE staining of abdominal lymph node biopsy under high magnification (magnified×40). (E) HE staining of abdominal lymph node biopsy under low magnification (magnified ×10).

We conducted a detailed examination of the patient. Her blood laboratory values after admission were as follows: white blood cells: 3.3 × 10^9^/L, red blood cells: 2.57 × 10^12^/L, hemoglobin: 72 g/L, and platelets: 65 × 10^9^/L. Coagulation function: a prothrombin time of 13.6 s and an international standardized ratio of 1.25. The following biochemical values were determined: albumin, 22 g/L; globulin, 30 g/L; creatinine, 330 μmol/L; and 24‐h urinary protein, 2512 mg. The results of PET/CT revealed the following: 1. Multiple lymphadenopathy in the parainferior vena cava, hilar, retroperitoneal, and perihilar regions; increased FDG metabolism; and splenomegaly, with suspicion of lymphoma; and 2. lymphomatous infiltration of the right upper humerus, left sacrum, bilateral ilium, and bilateral upper femur (Figure 1A,B). To confirm the diagnosis, the patient's left kidney and abdominal lymph nodes were subsequently biopsied. The pathological results of the kidney revealed membranous proliferative glomerulonephritis (MPGN), a moderately to severely widened glomerular mesangial area, increased mesangial cell proliferation, an increased mesangial matrix, increased diffuse capillary cavity cells, basement membrane thickening, and segmental double‐track sign formation (Figure 2A–C). Pathological analysis of the abdominal lymph nodes revealed highly invasive B‐cell non‐Hodgkin's lymphoma (Figure 2D,E); this analysis combined with immunohistochemistry indicated a CD5‐positive DLBCL (non‐germinal center B‐cell‐like lymphoma source). Immunohistochemistry revealed the following tumor cell types: CD20+, PAX5+, CD3‐, CD5+, CD10‐, CD30‐, CyclinD1‐, SOX11‐, and Bcl‐2+ (approximately 60%), C‐myc + (approximately 40%), MUM‐1+, a small amount of Bcl‐6+, and Ki‐67+ (approximately 85%). In situ hybridization revealed EBER negativity.

## 4 Results

On the basis of the patient's clinical manifestations combined with her physical examination and pathological results, she was finally diagnosed with DLBCL stage IV group B and NS. Next, six cycles of R‐CHOP (rituximab, cyclophosphamide, doxorubicin, vincristine and prednisone) were given. After treatment, the patient's clinical manifestations improved significantly. The biochemical values were as follows: albumin, 41.5 g/L; and creatinine, 47 μmol/L. The patient's blood parameters were as follows: white blood cell count, 6.3 × 10^9^/L; hemoglobin, 104 g/L; and platelet count, 332 × 10^9^/L. Abdominal CT examination showed that the lymph nodes around the original inferior vena cava, hilar, retroperitoneal, and splenic hilum were significantly smaller than before (Figure 1C,D). In summary, as the lymphoma entered a complete remission period, the symptoms of NS were significantly relieved.

## 5 Discussion

DLBCL is a malignant tumor that originates from mature B cells and has highly heterogeneous clinical features and prognoses. DLBCL is the most common pathological type of non‐Hodgkin lymphoma (NHL), accounting for 25% of all NHLs [5, 6]. DLBCL exhibits strong heterogeneity in terms of clinical characteristics, laboratory indicators, immune phenotypes, and responsiveness to treatment regimens [5]. Common symptoms include enlarged lymph nodes, fever, night sweats, reduced body mass, fatigue, etc. Clinically, DLBCL rarely invades the kidneys. Data and literature describing the clinical processes and outcomes of patients with DLBCL and renal involvement are very limited [2].

NS is a common renal disease characterized by high proteinuria (urine protein > 3.5 g/d), hypoalbuminemia (serum albumin < 30 g/L), edema, and hyperlipidemia [7]. NS can be divided into primary and secondary forms. Among the factors associated with secondary NS, malignant tumors account for approximately 10% of cases, of which lung and gastrointestinal tumors have the highest incidence, while breast cancer, renal cell carcinoma, thyroid cancer, multiple myeloma, and NHL are also observed [8, 9]. The relationship between malignant tumors and NS was first proposed by Lee et al. in 1966 [8]. Since then, malignant tumor‐associated NS has attracted the attention of clinical physicians as a common secondary cause of NS. Villa et al. reported that out of 2656 patients with DLBCL, 55 (2%) had invasion of the kidneys. In addition, the researchers found that patients with DLBCL and renal involvement had a high incidence of recurrence in the central nervous system (CNS) [2]. Nicola et al. evaluated 22 patients with DLBCL and renal involvement and found that although these patients had received a curative regimen containing rituximab, most of them were in the late stage of the disease, the prognosis was poor, and there was CNS involvement and recurrence [10].

The pathological changes in the kidneys of the patient mainly involved MPGN‐like changes and crescent formation, no lymphoma infiltration, and sensitivity to R‐CHOP treatment. Keisuke et al. reported a case of NS with DLBCL [11]. Compared with our patient, there are several differences. First, the cause of NS in their patient was membranous nephropathy, whereas our patient had membranous proliferative glomerulonephritis. Second, Keisuke's patient had DLBCL infiltration in the kidney, whereas our patient had no DLBCL infiltration in the kidney. Finally, the treatment options were also different. Considering the adverse reactions of renal failure and hypoproteinaemia, Keisuke et al. first used the CHOP regimen and then rituximab. The patient received < 50% of the chemotherapy dose in the first cycle of treatment. In contrast, our patient's condition was relatively mild, and when the R‐CHOP regimen was used, the effect was improved, and there were no adverse reactions. The case reported by Kareem et al. is somewhat similar to ours, and the patient's kidney was not directly affected by the lymphoma [3]. Many immune‐related genes, such as CRP, JAK1, and TANK, play a role in DLBCL. Moreover, the abnormal expression of these genes will also affect other organs and cause abnormal reactions [12]. Renal involvement is closely related to the progression of lymphoma [13]. In addition, the patient in our case was also CD5‐positive (CD5+) DLBCL. About 5%–10% of DLBCL express CD5, which makes CD5 DLBCL a rare subgroup. In addition, patients usually have advanced disease, and the incidence of CNS recurrence and bone marrow involvement is high. Studies have found that rituximab can improve the OS of CD5+ DLBCL patients, but does not reduce the CNS recurrence rate. Patients with CD5+ DLBCL need more effective CNS prophylaxis. Because the patient refused bone marrow biopsy, we could not know whether his CNS and bone marrow were involved, and could only ask him to follow up regularly.

The clinical manifestations and pathological changes associated with kidney damage caused by DLBCL are diverse, making it difficult to distinguish DLBCL from primary and secondary kidney diseases in clinical practice [14, 15]. When the following situations occur, care should be taken to rule out DLBCL‐related kidney damage: (1) obvious hematopoietic system damage, such as anemia or an abnormal increase or decrease in white blood cells that does not match the renal function; (2) swelling of multiple superficial or deep lymph nodes; (3) specific damage to the skin and nasal mucosa; (4) the appearance of ANA, ANCA, or anti‐GBM antibodies, serum monoclonal light chains, or cryoglobulin that cannot be explained by typical systemic vasculitis or light chain deposition disease; (5) single lymphoid‐like cell infiltration with focal aggregation in renal tissue; and (6) mild glomerular lesions but significant lymphocyte infiltration in the peritubular capillaries of the glomerulus [16, 17, 18]. If the above conditions are clinically observed, the following examinations should be performed according to the patient's condition: bone marrow cell examination and bone marrow biopsy, lymph node biopsy, biopsy of specific areas (such as the skin, nasal mucosa, and intestinal mucosa), serum cryoglobulin examination, immunofixation electrophoresis, and immunohistochemical examination of T cells and B cells in renal tissue.

In conclusion, there are few DLBCL patients with NS as the main manifestation, and even this symptom can precede the typical manifestation of lymphoma, resulting in clinical missed diagnosis. Clinicians should pay attention to the diagnosis of NS etiology, especially for NS patients with poor treatment effect; clinicians should actively and comprehensively find the cause. For patients with renal damage combined with anemia, elevated globulin, elevated immunoglobulin, and lymph node enlargement, we should consider the possibility of lymphoma and improve renal biopsy, lymph node biopsy, blood, and bone marrow specimens in time.

## Author Contributions

**Hongrui Xiao:** conceptualization, data curation, formal analysis, investigation, methodology, project administration, writing – original draft, writing – review and editing. **Lei Ye:** writing – original draft, writing – review and editing. **Fenjuan Zhang:** conceptualization, data curation, writing – original draft.

## Ethics Statement

Approval was obtained from the Institutional Review Board of Lishui Central Hospital (No. 2023816). The procedures used in this study adhere to the tenets of the Declaration of Helsinki.

## Consent

Informed written consent was obtained from the patient for publication of this report and any accompanying images.

## Conflicts of Interest

The authors declare no conflicts of interest.

## Data Availability

The data are available for sharing.
